# Mild atopic dermatitis is characterized by increase in non-staphylococcus pathobionts and loss of specific species

**DOI:** 10.1038/s41598-024-74513-2

**Published:** 2024-10-10

**Authors:** Lize Delanghe, Ilke De Boeck, Joke Van Malderen, Camille Nina Allonsius, Tim Van Rillaer, Peter A. Bron, Ingmar Claes, Margo Hagendorens, Sarah Lebeer, Julie Leysen

**Affiliations:** 1 Department of Bioscience Engineering, University of Antwerp, Groenenborgerlaan 171, Antwerpen, B-2020 Belgium; 2 University Hospital Antwerp, Department of Pediatrics, University of Antwerp, Wilrijkstraat 10, Edegem, B-2650 Belgium; 3 University Hospital Antwerp, Department of Dermatology, University of Antwerp, Wilrijkstraat 10, Edegem, B-2650 Belgium; 4 YUN NV, Galileilaan 15, Niel, B-2845 Belgium

**Keywords:** Bioinformatics, Sequencing, Applied microbiology, Microbial communities, Skin diseases

## Abstract

Atopic dermatitis is the most common inflammatory skin condition with a severe negative impact on patients’ quality of life. The etiology of AD is complex and depends on age, genetics, the immune system, environmental factors, and the skin microbiome, with a key role for pathogenic *Staphylococcus aureus* in the development of severe AD. However, the composition of the skin microbiome in mild AD is understudied. Here, using metagenomic shallow shotgun sequencing, we showed that mild AD lesions did not show a significant difference in the diversity of the skin microbiome compared to samples from non-AD patients and that the relative abundance of *S. aureus* did not differ in these mild AD lesions. However, when we assessed other taxa, *Mycobacterium ostraviense*, *Pedobacter panaciterrae_A* and four *Streptomyces* species were identified with higher abundances in mild AD lesions and species of 15 genera were decreased in abundance. The highest fold decreases were observed for *Paracoccus marcusii*, *Microbacterium lacticum*, *Micrococcus luteus*, and *Moraxella sp002478835.* These microbiome compositional insights are a first step towards novel microbiome-based diagnostics and therapeutics for early intervention at the stage of mild AD and provide a path forward for the functional study of species involved in this often-overlooked patient population.

**Supplementary Information:**

The online version contains supplementary material available at 10.1038/s41598-024-74513-2.

## Introduction

Atopic dermatitis (AD) is a chronic inflammatory skin condition that affects approximately 20% of children and 3% of adults in Western countries^1,2^. The clinical diagnosis of AD is made based on the Hanifin and Rajkia criteria and the symptoms are characterized by flares of itchy, eczema lesions, and red and dry skin^3^. AD can manifest in a different grade of severity, ranging from ‘mild’ to ‘severe’ which can be examined by validated severity assessment tools such as the Scoring AD (SCORAD) index or Eczema Area and Severity Index (EASI)^4^. However, the differences in pathophysiology between ‘mild’ and ‘severe’ AD are not well understood and depend on the site of the lesion.

AD usually starts early during childhood and is a common first symptom in the so-called “atopic march”, describing the successive development of AD, allergic rhinitis, and asthma in atopic children^5^. The symptoms, which often cause insomnia and social insecurity, have a large impact on patients’ quality of life, even for mild AD symptoms^6,7^. In addition, the high prevalence creates a significant burden on health facilities^8,9^. The etiology of this skin condition is very complex, with the appearance influenced by genetic and immunological mechanisms, as well as environmental factors^1^. Important genetic risk factors include mutations in genes encoding proteins for skin barrier integrity, such as loss-of-function mutations in the filaggrin gene^10^, or variants of several genes primarily involved in immune pathways, such as Interleukin-4 receptor (IL-4R), Interleukin-18 (IL-18), and Interleukin-31 (IL-31)^11–13^. The complex immune dysregulation in AD includes an important role of T-helper (Th)-2 inflammation, with IL-4 and IL-13 involved in both acute and chronic AD lesions, and Th-1 inflammation, with interferon-gamma involved in chronic AD lesions^14,15^. In addition, skin irritants and allergens; climate factors, such as temperature, UV radiation and humidity; maternal exposures during pregnancy, such as smoking, probiotic intake and diet^15^ can all be involved, depending on the geographical region studied. Unfortunately, the latter is not always properly reported in studies on AD.

In addition to the factors described above, the skin microbiome is increasingly considered as another factor influencing the etiology of AD, as different studies highlight that disturbances in the microbial communities on the skin can lead to various skin disorders^16,17^. In addition to the importance of the entire microbial skin communities, *S. aureus*has been described as a major pathogen for AD for decades^18^. For example, using metagenomic shotgun sequencing, one study with 11 children with moderate-to-severe AD versus 7 healthy controls (ages 2–18, United Kingdom) has found that flare-ups are associated with colonization by *S. aureus*^19^. Another study with 12 children with moderate AD versus 11 healthy controls (ages 2–15, USA) confirmed these findings using a full-length 16 S gene sequencing approach^20^. Both studies also found associations between the disease severity and the abundance of this species, with significantly higher *S. aureus* relative abundances in severe AD. However, *S. aureus*does not seem to be the single microbiological cause of AD, since the disease severity appears also to be associated with a reduced species richness and evenness in these two studies^19,20^. Furthermore, in a study in adults with moderate-to-severe AD, a reduced alpha diversity was also observed, which included a reduced evenness of the microbial population mainly driven by *S. aureus*, while the overall richness was not reduced^21^. When looking at studies also including mild AD patients, differences in beta- and alpha diversity between those patients and healthy controls appear not so apparent: the observed microbiome changes are mainly focused on specific taxa, such as the genera *Staphylococcus* and *Streptococcus*^22,23^. So far, studies investigating the differences between the healthy and AD skin microbiome have included a limited age group (children or adults) and mostly focused on moderate-to-severe AD^19–21,24,25^, while excluding AD patients with milder manifestations of disease which make up to 60% of the patient population^26^. To gain better insights into the taxa important for the microbiome changes in mild AD manifestations, we need more studies including these patients and using next generation sequencing techniques, such as metagenomic sequencing, that can profile their skin microbiome up to species level.

In this study, we aimed to characterize the skin microbiome in a Belgian cohort of 38 AD patients versus 49 healthy subjects to identify skin microbiome biomarkers, especially for mild AD which is currently underexplored in skin microbiome studies. Patients were recruited during standard follow-up consultations at our university hospital. We included AD patients with mild lesions (*n* = 28) or moderate-to-severe lesions (*n*= 6) at any body site, of a relatively wide age range, grouped in different age groups: 0–3 years, 3–6 years, 6–12 years, 12–18 years and 18 years and older. The severity of the AD lesions was determined by using a local scoring system based on the Eczema Area and Severity Index (EASI)^27^. All skin swabs were analyzed via metagenomic shallow shotgun sequencing to identify the skin microbial composition up to species level in a cost-effective way.

## Results

**Overall skin microbiome diversity is determined by the severity of the AD lesion and age of the patient**.

We first explored whether the severity of AD lesions - stratified here in mild and moderate-to-severe AD by EASI scoring – distinctly impacted the overall diversity of the skin microbiota compared to healthy skin and whether this depended on age of the participants and physiological skin site. 94% of the samples passed the quality control pipeline and high-quality microbiome profiles were obtained from 85 participants of our cohort, including 45 healthy controls, 28 mild AD patients and 6 moderate-to-severe AD patients.

**Fig. 1:**
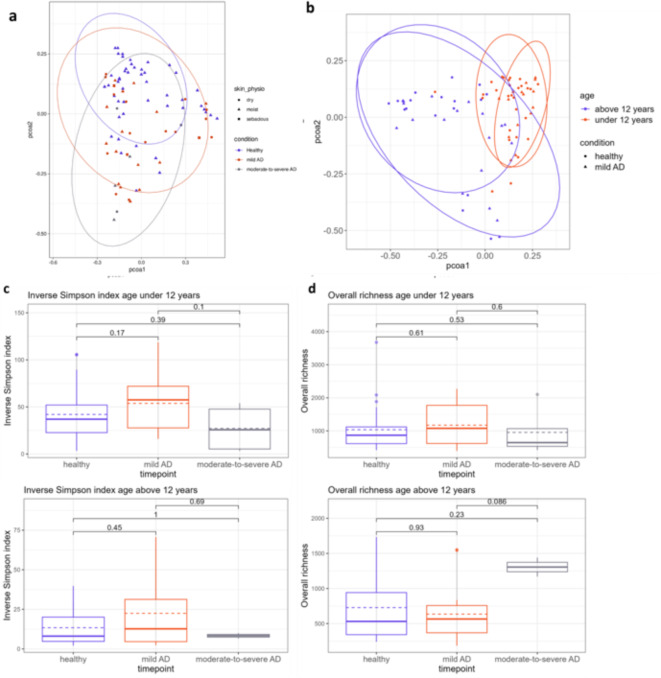
Associations between atopic dermatitis and skin microbiome diversity. (a) Principal Coordinates Analysis (PCoA) plot visualizing the beta diversity of all skin samples, colored by condition and different shapes show the physiological skin sites. Ellipses show a 95% confidence interval for each condition. Ellipses show a 95% confidence interval for each condition. (b) PCoA plot visualizing the beta diversity of healthy and mild AD samples, colored by age. Ellipses show a 95% confidence interval for each age group. (c) Inverse Simpson index for skin samples separated in age groups below and above 12 years old, grouped by condition. (d) Overall richness for skin samples separated in age groups below and above 12 years old, grouped by condition. Statistics were performed using the Mann-Whitney-Wilcoxon test.

Analyzing beta-diversity, or the diversity of taxa between samples, was done by calculating Bray-Curtis dissimilarity (at species level) and visualizing the samples in a Principal Coordinates Analysis plot (PCoA) based on condition (healthy, mild, or moderate-to-severe AD), age and the skin site of the sampled areas. For investigating the effect of age, a threshold was set at 12 years old based on our previous research on the impact of age on the healthy skin microbiome where we could identify the highest impact of age on skin microbiome diversity and composition when comparing healthy controls below and above 12 years old. When exploring the effect of these factors on bacterial community with a PERMANOVA (including the factors age and condition, in this order), 9.8 and 7.5% of the whole-community variation was explained by respectively age (below and above 12 years old) and condition (AD or healthy). When exploring the effects of age and condition on the bacterial community with Adonis, beta-diversity was significantly different based on both age (R^2^ value 0.0985, *p*< 0.001) and condition (R^2^ value 0.0747, *p*< 0.001) with a higher R^2^ value for age. Although samples from AD lesions were derived from skin sites with a different biogeography and physiology (moist, sebaceous and dry sites), they did not cluster based on skin site or physiology in our dataset (PERMANOVA including the factors skin site and skin physiology, *p* = 0.221 and *p* = 0.153 respectively). Therefore, for subsequent analyses, all AD samples were used for comparison with the healthy controls (Fig. 1a, Figure S1).

When specifically looking at the mild AD lesions compared to healthy controls, these samples could not be clearly separated from healthy controls (Fig. 1a), indicating that their overall composition (at beta diversity level) was not different. However, for these mild-AD samples, the AD condition did still explain 4.6% (*p* < 0.001) of the variation (Fig. 1b). Nevertheless, age accounted for a larger part of the variation (10.9%, *p* < 0.001), suggesting that age is the most decisive factor in shaping the skin microbiome in mild AD.

Next, we investigated the alpha diversity (or the diversity within samples) of samples from specific severities of AD lesions. Because of the high impact of age below and above 12 years old on the beta diversity, we analyzed alpha diversity separately for these two age groups. No significant differences were observed for the Inverse Simpson index, which takes the evenness of taxa abundance in a sample into account (Fig. 1c). When alpha diversity was measured by overall richness (the number of taxa in a sample), we also observed no significant differences (Fig. 1d). This analysis indicated that the number of species on the skin was similar for AD patients and healthy controls. In contrast to the AD severity, alpha diversity was significantly impacted by the age of AD patients (below and above 12 years old) (Figure S2).

### Core abundant and prevalent skin genera affected in mild AD lesions

**Fig. 2:**
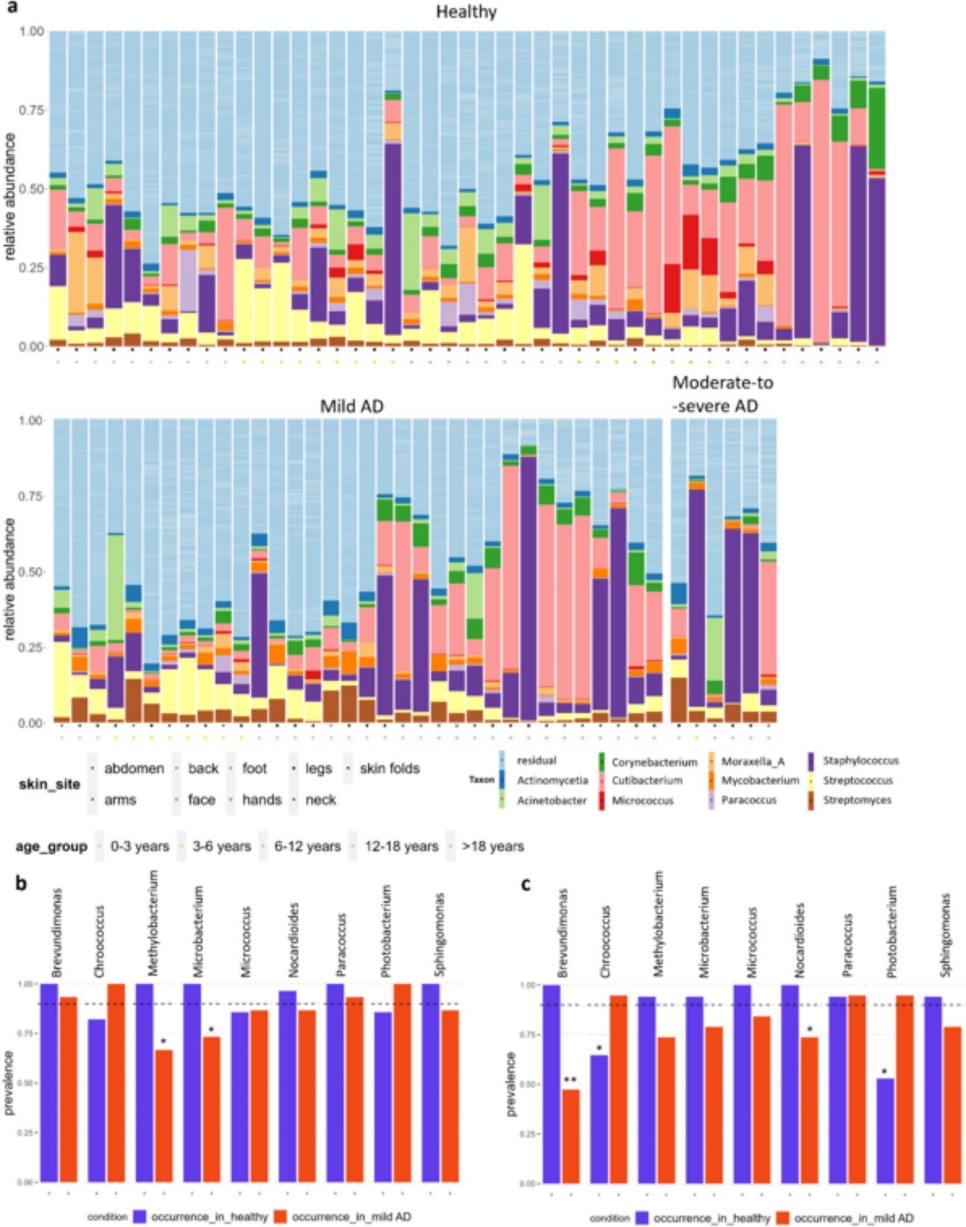
The core genera of the skin microbiome are different in mild AD compared to healthy skin (a) Skin microbiome profiles of all healthy and AD samples, that passed quality control after sequencing, at genus level. Each bar represents the microbial profile of one individual and the 11 most abundant taxa are shown (the rest is grouped in residual). Colors and shapes refer to the age of the participant and the skin site of sampling. (b) Barplot showing the prevalence of genera in the healthy compared to mild AD samples, for all participants below 12 years old. (c) Barplot showing the prevalence of genera in the healthy compared to mild AD samples, for all participants of 12 years and older. Significant differences were calculated using a fisher test. ** *p* < 0.001, * *p* < 0.05.

Since no clear impact of mild AD manifestation on skin diversity at the lesion site was observed, we subsequently investigated whether specific taxa were different between AD skin and healthy skin. Hereto, while we acknowledge that rare taxa can also be of functional importance, we first looked at the differential abundance of the 11 most abundant taxa in both healthy and AD samples, identified here as core abundant taxa: the class *Actinomycetia* (32%), and the *genera Acinetobacter* (3.5%), *Corynebacterium* (2.7%), *Cutibacterium* (13.5%), *Micrococcus* (0.9%), *Moraxella* (1.9%), *Mycobacterium* (1.9%), *Paracoccus* (1.1%), *Staphylococcus* (19.4%), *Streptococcus* (5.3%) and *Streptomyces* (3.6%) (Fig. 2a). Because of their higher abundance, these genera could be considered successful in adapting to the skin habitat. Consequently, their difference in dynamic abundance based on AD manifestation could give information on the changing ecosystem upon AD disease. Our analysis showed that the genera *Mycobacterium* (*p* = 0.0012) and *Streptomyces* (*p* = 2.2 × 10^-5) are of particular interest, because they showed a 2.2 to 2.9-fold increased relative abundance respectively in mild AD compared to the healthy controls (Figure S3). These genera were also significantly increased in moderate-to-severe cases by respectively 2.2 (*p* = 0.0097) and 4.2-fold (*p* = 2.5 × 10^-4) (Figure S3).

In addition, we looked at the taxa that were decreased in the AD lesions, because these can include taxa that could have potential protective functions against AD or could be taxa that are more tolerant to the specific stress conditions of AD disease. The abundances of *Moraxella*,* Micrococcus*, *Acinetobacter*, and *Paracoccus* were significantly decreased in both AD groups as compared to healthy samples. Additionally, *Corynebacterium* (*p* = 0.04) and *Cutibacterium* (*p* = 0.034) were significantly decreased, specifically in the moderate-to-severe compared to healthy samples (Figure S3). Based on age, the largest differences were observed when comparing samples below and above 12 years old, with a higher abundance of *Cutibacterium* and lower abundance of *Streptococcus* in participants of 12 years and older (Fig. 2a). Of interest, the specific physiological skin sites sampled within AD patients did not impact the relative abundance of the 10 most abundant genera, which is in line with the beta diversity observation (Fig. 1a).

Next to relative abundance, the prevalence of genera also provides relevant information on the skin microbiome and how it could be affected by AD. Taxa with a high prevalence, but not necessarily a high abundance, can exert important keystone microbiome functions. In the group of healthy and mild AD samples, 27 and 22 genera had a prevalence of at least 80%, respectively (Figure S4). In the group above 12 years old, two of these genera did show a significantly lower prevalence in the healthy control samples: *Chroococcus* (*p* = 0.037) and *Photobacterium* (*p* = 0.0061) (Fig. 2c). This could suggest that these taxa have potential keystone pathogenic functions for mild AD. In contrast, four genera displayed a significantly higher prevalence in healthy samples: *Methylobacterium* (*p* = 0.0031) and *Microbacterium* (*p* = 0.011) for the samples under 12 years old, and *Brevundimonas* (*p* = 0.00044) and *Nocardiodes* (*p* = 0.047) for the samples above 12 years old (Fig. 2b and c). Of interest, *Brevundimonas* did show a 100% prevalence in healthy samples, and only 47% in mild AD samples in this age group. These taxa could thus represent the loss of possible keystone protective microbiome functions.

## Altered abundance of potential pathobiont species and decrease of endogenous microbiota members in mild AD

After observing differences in the relative abundance and prevalence of core genera, we explored these taxa further up to species level, because this taxonomic level provides more functional information.

**Fig. 3:**
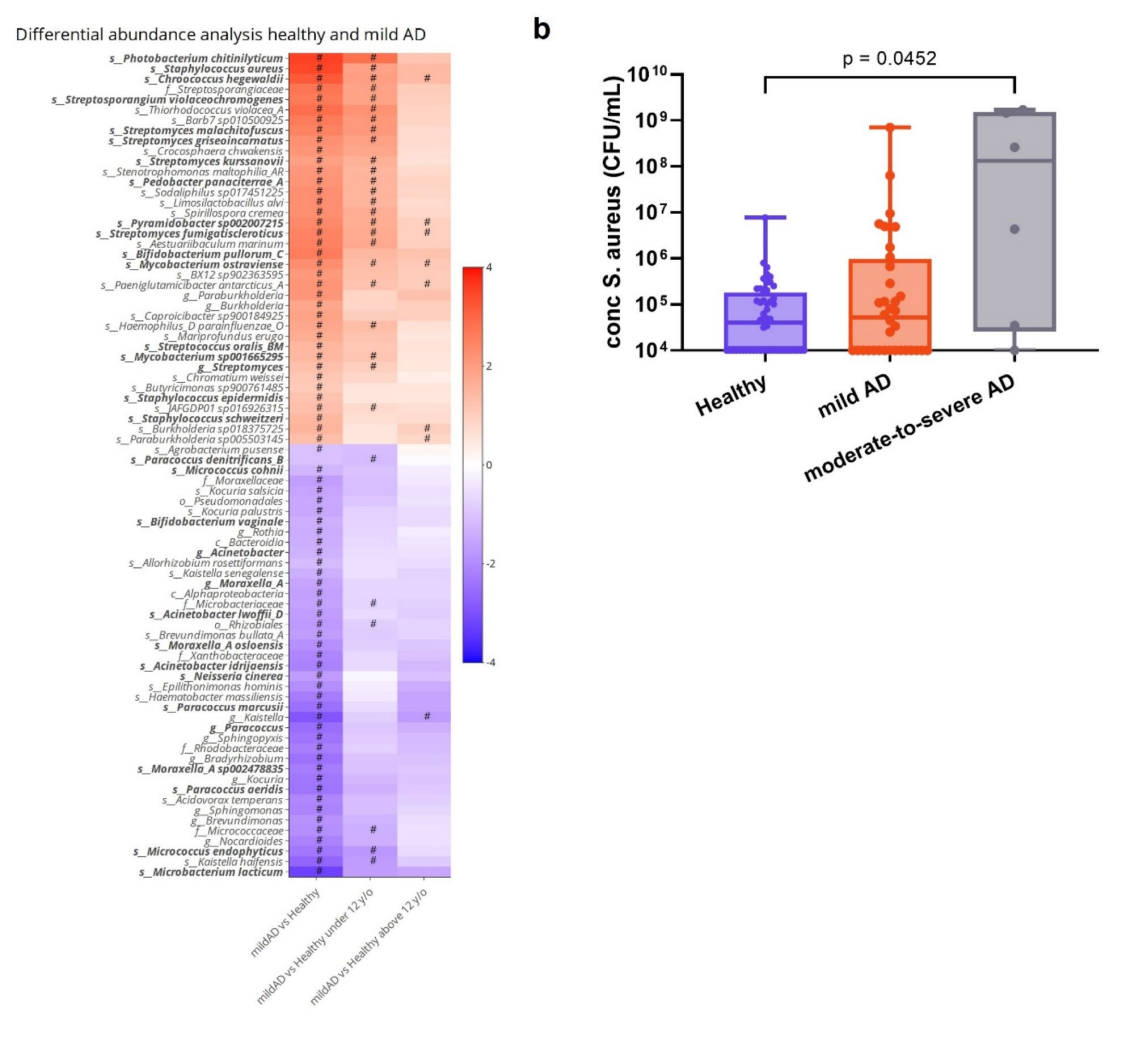
The effect of host variables on the relative abundances of skin taxa (a) Differential abundance analysis for mild AD patients compared to healthy participants using Maaslin2 (Materials and Methods). Cell shading represent the effect sizes of differences in skin taxa relative abundance based on health condition (healthy or mild AD) and age (below and above 12 years old). Red and blue represent an increase and decrease in relative abundance of the indicated taxa, respectively. Asterisks indicate FDR adjusted significant p-values < 0.05. Indicated in bold are the genera with a high abundance and/or prevalence. Taxa were ordered according to a hierarchical clustering with complete linkage and a correlation distance, to group similar responses together.

At genus level, no significant differential abundance levels were observed for *Staphylococcus* between mild or moderate-to-severe AD lesions and healthy skin samples. However, at species level, S*taphylococcus aureus* and *Staphylococcus epidermidis* displayed a significant higher relative abundance on mild AD lesions compared to healthy skin, on average 40.2 and 2-fold higher respectively (Fig. 3a). For moderate-to-severe lesions, the increase of *S. aureus* was even more prominent, with a 368.3-fold increase. For *S. epidermidis*, a 3-fold decrease was observed in moderate-to-severe AD lesions compared to healthy skin. When we explored this increase of *S. aureus* based on absolute abundance, quantitative PCR showed an increasing trend for AD samples, but this not significant for mild lesions (*p* = 0.9822) and only significant for moderate-to-severe lesions (*p* = 0.0452) (Fig. 3b). This absolute abundance of *S. aureus* was also analyzed separately for two different age groups (below and above 12 years old) and showed the same trend, with a significant increase for moderate-to-severe AD in the age group above 12 years old (Figure S7).

We then decided to focus on the non-staphylococcal potential pathobionts based on differences in relative abundances between the healthy controls and AD patients. We found that *Mycobacterium ostraviense*,* Chroococcus hegewaldii*,* Pyramidobacer sp002007215*, *Pedobacter panaciterrae_A*,* Streptosporangium violaceochromogenes* and four different *Streptomyces* species (*S. griseoincarnatus*, *S. kurssanovii*,* S. malachitofuscus* and *S. fumigatiscleoriti*s) showed at least a 3-fold higher relative abundance on mild AD lesions compared to healthy skin (Fig. 3a). The increase of *M. ostraviense*,* C. hegewaldii*,* P. sp00200715* and *S. fumigatiscleroticus* even remained significant for both age groups separately (below and above 12 years old), with a lower number of samples.

Finally, we explored which endogenous skin species showed a significantly lower abundance in mild AD lesions compared to the healthy samples. In total, a reduction in potentially protective species of 15 different genera was observed (Fig. 3a). At species level, the highest fold decreases were calculated for *Paracoccus marcusii* (21.7-fold), *Microbacterium lacticum* (6-fold), *Micrococcus luteus* (5.3-fold), *Moraxella sp002478835* (4.1-fold), *Micrococcus endophyticus* (3.6-fold), *Acinetobacter junii* (3.2-fold), *Lawsonella clavelandensis* (2-fold) and *Corynebacterium durum* (1.4-fold).

(b) qPCR for absolute abundance of *S. aureus.* Statistics were performed with Kruskal-Wallis test with Dunn’s multiple comparisons test against the healthy samples.

## Discussion

In this study, using shallow metagenomic shotgun sequencing, combined with qPCR, we uncovered novel differences at species level in the skin microbiome of AD lesions dependent on the severity, with the identification of novel possible biomarkers for mild AD.

The abundance of the canonical species *S. aureus*is often positively associated with AD disease severity^19,28,29^. We included both mild and moderate-to-severe AD patients to be able to compare *S. aureus* abundance in both groups. This comparison made it possible to suggest other microbial biomarkers specifically for mild AD patients, instead of focusing on *S. aureus* only in this patient group. We found that the absolute abundance of *S. aureus* was not significantly increased on mild AD lesions, both when analyzing all samples together and separated in two age groups (below and above 12 years old). At the relative abundance level, we did find a stepwise increase in *S. aureus* levels on mild AD lesions. However, this relative abundance of *S. aureus* on mild AD lesions in our study (2.4% +/- 4.8) was much lower than in previous studies with moderate-to-severe AD patients, possibly due to differences in AD disease severity. For example, Kong et al. calculated a *S. aureus*relative abundance of 65% on moderate AD lesions^20^. We could identify *Mycobacterium ostraviense*,* Chroococcus hegewaldii*,* Pyramidobacer sp002007215*, *Pedobacter panaciterrae_A*,* Streptosporangium violaceochromogenes* and four *Streptomyces* species (*S. griseoincarnatus*, *S. kurssanovii*,* S. malachitofuscus* and *S. fumigatiscleoriti*s) as species with a higher relative abundance on mild AD lesions. These mild AD-specific biomarkers identified in our study, which were so far underexplored, potentially enables earlier detection and treatment. A more in-depth evaluation of the functional properties of these less-explored potential pathobionts should be carried in the near future, since solely microbiome based associations do not necessary imply causality^30^. Such functional studies are required, because an enhanced abundance in disease conditions such as AD could also reflect that these species are just more tolerant to and more metabolically independent upon the stressful skin conditions of the disease, without the species themselves expressing virulence factors, as has been previously reported for other microbiome studies^31^.

We also investigated whether the observed differences between the severity of the AD lesions and healthy controls could be biased by medication use or other host factors. The use of oral and topical antibiotics, topical corticosteroids, antihistaminic and allergies did not impact the differential abundances of the different taxa (Figure S5). We only found that oral antibiotic intake within 4 weeks before sampling led to a higher abundance of *Staphylococcus nepalensis* on AD lesions (Figure S3).

While a lot of attention in microbiome studies is paid to potential pathobionts, we also explored taxa that were decreased in mild AD samples based on their relative abundance, because these taxa might hold potential protective functions and offer novel therapeutic options. *Paracoccus marcusii*, *Microbacterium lacticum*, *Micrococcus luteus*, *Moraxella sp002478835*, *Micrococcus endophyticus*, *Acinetobacter junii*, *Lawsonella clavelandensis* and *Corynebacterium durum* were particularly decreased on mild AD lesions. Of these identified taxa, the decreased abundance of *Moraxella sp002478835* in AD lesions is very interesting as a possible biomarker since *Moraxella* was found as a member of the core skin microbiome of our healthy population (Fig. 2a). However, the role of *Moraxella* is not clearly beneficial on all body sites in AD or for all species, since previous research has found that *Moraxella*in the nares of children is positively associated with AD disease severity^32^. *Micrococcus endophyticus* and *M. luteus* could be of more interest to explore as future microbial therapeutics for AD. *M. luteus*is a well-known skin commensal that is also often found in fermented foods and the environment^33^. Of note, *M. luteus*Q24 strain was even recently included as topical skin probiotic in a clinical trial and showed improvement in the skin appearance of healthy individuals^34^. In addition, different *Corynebacterium* species are worth further exploring as potential skin probiotics, such as *C. durum*, because they were reduced on the AD lesions in our study. Of note, reductions in *Corynebacterium*taxa were previously related with AD^18^. *Corynebacterium*even showed to be increased together with an increase in overall microbial diversity after dupilumab therapy, a monoclonal antibody to the alpha subunit of IL-4 and IL-13 receptors^35^. Nevertheless, *Paracoccus marcusii* and *Microbacterium lacticum* could have most potential as potential protective species, because of the high fold change in relative abundance for *P. marcusii* (21.7-fold) and the significantly lowered prevalence and relative abundance of *M. lacticum* in our mild AD samples. In our study and in previous research, *Paracoccus*was identified to be part of the 10 most abundant genera on healthy skin^36^. *P. marcusii*is known as a natural producer of astaxanthin, a xanthophyll carotenoid, which has potential health-promoting effects in the prevention or treatment of various diseases^37,38^. *Microbacterium*was previously identified to be a member of the healthy skin microbiome, for example in the inner elbow^39^and the abundance of this genus was found to be decreased in vitiligo skin lesions^40^. In addition, both *Paracoccus* and *Microbacterium*were found to increase in abundance during the skin wound healing process^41^. Their high relative abundance and/or prevalence in healthy samples and decreased abundance in mild AD lesions, indicates that they might contain protective functions that are lost in AD. To the best of our knowledge, their protective role is fully unexplored for the skin and requires documentation in future functional studies. A good example of such protective role for skin commensals is the study of van der Krieken et al. (2023), in which they elucidated the protective function of Gram-positive anaerobic cocci, species that are less abundant on filaggrin deficient skin, via induction host-defense molecules and immune-cell activation^42^. Such functional studies are indeed necessary, because their altered prevalence upon disease could also merely reflect metabolic dependencies and enhanced sensitivity to the stressful conditions of the disease^31^, as already indicated above for opposite observations for the potential pathobionts.

It should be noted that merely the decreased abundance or prevalence of a species in disease is insufficient to determine whether a species is potentially protective and whether the species could be further developed into a skin probiotic. Hereto, in line with the conceptual idea behind the Koch postulates for pathogens^43^, the potential protective function of taxa should also be first substantiated in different mechanistic and relevant functional models. Such functional studies will have to include a thorough screening for safety: absence of virulence properties and lack of antibiotic resistance markers on mobile elements, similarly also for all probiotics^44^. Therefore, the development of skin probiotics from taxa that do not belong to a biosafety level 1 or have a ‘generally recognized as safe’ (GRAS in US) or ‘qualified presumption of safety (QPS state in EU) will be more complex than taxa that belong to traditional probiotic genera such as lactobacilli^45^. Nevertheless, the data presented in this study show that it is worth exploring these non-typical taxa. Similarly, at pathobiont level, our shotgun data presented here point out that it is of interest to further explore the potential skin pathogenic role of *Mycobacterium ostraviense*,* Chroococcus hegewaldii*,* Pyramidobacer sp002007215*, *Pedobacter panaciterrae_A*,* Streptosporangium violaceochromogenes*, and four *Streptomyces* species.

Finally, we also want to highlight some limitations of our study. This study was designed based on the routine follow-up of AD patients at our university hospital, where it was the first time that microbiome swabs were added to the routine consultation. Therefore, the data presented here were based on a single sample for each patient. For further research, it would also be interesting to collect samples before and after flare-ups to capture spatial and temporal variations more adequately. This would need more complex study design and planning since timepoints of flare-ups are difficult to predict. In addition, higher resolution taxonomic profiling (up to strain-level, and functional annotations of bacterial genes) with deep metagenomic shotgun sequencing would be of high value for a follow-up study to validate the hypotheses found in this work. Specifically for our shallow metagenomic shotgun sequencing approach, it would also be of benefit to include positive controls, in addition to the negative controls that were included, to allow better of the quality of the sequencing data. Nevertheless, this study shows that microbiome analysis is of high interest to include in investigational dermatology to improve the development of new and efficient therapies with multifactorial working mechanisms and better prediction of responders and non-responders based on their skin microbiome profile.

## Conclusion

Our shallow shotgun sequencing study identified several new potential microbiome biomarkers for mild AD based on their altered prevalence and relative abundance. The identified specific species can now be further explored as potential beneficial (e.g. *Paracoccus* and *Microbacterium* species) and pathobiont (e.g. *Mycobacterium*,* Pedobacter* and *Streptomyces* species) skin microbiome members for AD. This study also paves the way for future mechanistic studies to unravel the role of these species on the human skin, which can lead to the design better diagnostic and therapeutic approaches for AD patients.

## Materials and methods

### Human subjects

Human subjects, age from 6 months to 70 years old, were recruited via email for healthy subjects and via the Antwerp University Hospital UZA (Departments of Pediatrics and Dermatology, Prof. Dr. Hagendorens and Dr. Leysen) for AD patients. Period of recruitment was from January to March 2021. In total, 49 healthy participants (average age 11.9 +/- 11.8) and 34 AD patients (average age 20.4 +/- 17.0) participated in the study. All participants were recruited in a highly urbanized region in Antwerp, Belgium. Healthy controls and AD patients were included starting from the age of 6 months. For the healthy controls, exclusion criteria were self-reported absence of atopic dermatitis symptoms or other skin conditions, and the use of topical corticosteroids and/or antibiotics within two weeks and use of oral antibiotics within four weeks before sampling were reported. For AD patients, the diagnosis of AD was made by the responsible medical professionals of this study based on the Hanifin and Rajka criteria^3^. Inclusion criteria for the patients were the diagnosis of AD and the presence of at least one clear AD lesion (mild, moderate or severe). Exclusion criteria were: (i) no other skin conditions than atopic dermatitis, (ii) no immunotherapy and (iii) no immunodeficiency diseases.

One AD lesion for each patient was selected for sampling and samples were taken in flare state. The severity of the AD lesions was determined by using a local scoring system based on the Eczema Area and Severity Index (EASI)^27^. The EASI scoring was used for local scoring where erythema (0–3), oedema (0–3), lichenification (0–3) and scratching (0–3) was scored for the lesion, no area scoring was done. Mild lesions were defined if a maximum of one symptom was scored with 2. Moderate-to-severe lesions were defined if at least two out of four symptoms were scored with 2 or at least one symptom was scored with 3. This study was conducted in accordance with the Declaration of Helsinki and the Guideline for Good Clinical Practice. The study protocol was approved by the Ethics Committee of the Antwerp University Hospital/University of Antwerp (registration number B3002020000099, ClinicalTrials.gov Identifier: https://clinicaltrials.gov/study/NCT04771910. The written informed consent was obtained from all participants or their parents/legal guardian prior to sampling. Table S2 gives an overview of participant’s demographics.

## Sample collection

For AD patients, the skin swabs were taken by the clinicians or study coordinator. All healthy participants received a detailed instruction booklet to do self-sampling of skin samples in a standardized way. For children under 16, a parent or other caretaker helped with the sampling. Skin samples were collected with the eNAT swab (Copan, Brescia, Italy). For all healthy participants, swabs were taken in the elbow bend. For AD patients, swabs were taken from a selected AD lesion site. Skin samples were collected by turning around the swab while brushing the area for 30 s. Prior to sampling, the swab was soaked in sterile pre-moisture buffer (50mM Tris buffer [Ph 7.6], and 0.5% Tween-20). The swabs were stored in the eNAT buffer at 4 °C for a maximum of two weeks and at -20 °C for a maximum of 4 months until further processing. All samples were registered in Biobank Antwerpen (Belgium) (identifier BE71030031000). An overview of the sampled skin site and sampling data for each participant can be found in Table S1.

## Microbial DNA extraction

DNA was extracted with the DNeasy PowerSoil Pro kit (Qiagen, Hilden, Germany) according to the manufacturer’s instructions. Prior to the DNA extraction, all swabs were vortexed for 15–30 s. DNA concentration of all samples was measured using the Qubit 3.0 Fluorometer (Life Technologies, Ledeberg, Belgium) according to the manufacturer’s instructions. Of each sample, 30 µL of gDNA was used for metagenomic shotgun sequencing library preparation to obtain a minimum of 1ng gDNA.

### Quantitative PCR (qpcr)

qPCR was used to determine absolute bacterial DNA concentrations of *Staphylococcus aureus*in samples after microbial DNA extraction. qPCR was performed as described before^46^. The following PCR conditions were used: pre-incubation at 50 °C for 10 min., denaturation at 95 °C for 20 s., followed by 40 cycles at 95 °C for 15 s., and 60 °C for 30 s., with the melting curve at 95 °C for 15 s., 60 °C for 1 min., 95 °C for 15 s. Primers were designed based on published sequences and primer design for intercalating dyes was performed in PrimerQuest™ Tool and synthesized by Integrated DNA Technologies (IDT, Leuven, Belgium) (S.aureus_ecpB forward primer: ATCATCGCCATGACGTATACAA, S.aureus_ecpB reverse primer: GACACAACCAAACTCACACATC). Standard curves were used to estimate bacterial DNA concentration in the samples and derived from serially diluted DNA from an overnight culture of *S. aureus*. Bacterial concentration was determined by plating out a serial dilution on Brain Heart Infusion agar (Neogen, Lansing, USA). Visualization of qPCR data was performed in GraphPad Prism 9, using a nonparametric Kruskal-Wallis test with Dunn’s multiple comparisons.

### Metagenomic shallow shotgun sequencing

Library preparation for metagenomic shotgun sequencing was performed using the Illumina DNA Prep (Illumina, San Diego California, United States) according to the instructions of the manufacturer and as described before^46^. Dual-index paired-end sequencing was performed using a MiSeq Reagent Kit V2 (300 cycles) (Illumina) and MiSeq Desktop sequencer (M00984, Illumina).

### Bioinformatic analysis

Metagenomic shotgun reads were processed as described before^46^. Samples contained on average 151.870 high-quality reads per sample and 18.984 bacterial reads per sample. Parsing of Kraken2 output was implemented in R version 4.2.1. Further processing of the taxa table, sample annotations, data visualization and statistical analysis were performed in R version 4.2.1 using the in-house R package tidyamplicons, version 0.2.2 [publicly available at github.com/SWittouck/tidyamplicons]. Negative controls for DNA extraction and library preparation were taken. Microbial composition of the negative controls can be found in Figure S6. Quality control was performed by filtering low read samples with reads equal to or lower than the negative controls. 95.2% of the samples passed the quality control pipeline and we obtained high quality profiles of 45 healthy participants and 34 AD patients. Samples AHD010, AHD011, ADH034 and ADH037 were excluded because of too low reads.

For alpha diversity, two measures were used: (1) observed diversity or the number of taxa, and (2) the Inverse Simpson index as a measure also taken the degree of evenness of a community. Statistics for alpha diversity were done using the Mann-Whitney-Wilcoxon test. To measure the beta diversity, the Bray-Curtis dissimilarity was calculated and visualized by a Principal Coordinates Analysis plot (PCoA). The effect of different factors such as age or skin condition on bacterial community composition was determined with a permutational multivariate analysis of variance or PERMANOVA using the Bray-Curtis distance matrix. Maaslin2 was used with default parameters, meaning TSS normalisation and Log transformation with a Linear Model, as recommended by the user manual. Multiple testing correction was done using the Benjamin-Hochberg method and results on the heatmaps were filtered to at least one significant adjusted p-value per covariate and a minimum effect size of 1.

## Electronic supplementary material

Below is the link to the electronic supplementary material.

Supplementary Material 1

## Data Availability

Datasets related to this article can be found at https://www.ebi.ac.uk/ena/browser/view/PRJEB63144 , with identification PRJEB63144, hosted at ENA, EMBL.
