# Supplementary material for: Mild atopic dermatitis is characterized by increase in non-staphylococcus pathobionts and loss of specific species
Source: Sci Rep. 2024 Oct 10;14:23659. doi: 10.1038/s41598-024-74513-2 (PMC11467409; doi:10.1038/s41598-024-74513-2)
Supplement: Supplementary file 1 — Supplementary Material 1 [file 41598_2024_74513_MOESM1_ESM.docx]

## Supplementary information

**Supplementary tables**

**Table S1:** Sampled skin sites for all participants

| **ID_participant** | **condition** | **skin_site** |
| --- | --- | --- |
| ADH001 | Healthy | skin folds |
| ADH002 | Healthy | skin folds |
| ADH003 | Healthy | skin folds |
| ADH004 | Healthy | skin folds |
| ADH005 | Healthy | skin folds |
| ADH006 | Healthy | skin folds |
| ADH007 | Healthy | skin folds |
| ADH009 | Healthy | skin folds |
| ADH010 | Healthy | skin folds |
| ADH011 | Healthy | skin folds |
| ADH012 | Healthy | skin folds |
| ADH013 | Healthy | skin folds |
| ADH014 | Healthy | skin folds |
| ADH015 | Healthy | skin folds |
| ADH016 | Healthy | skin folds |
| ADH017 | Healthy | skin folds |
| ADH018 | Healthy | skin folds |
| ADH019 | Healthy | skin folds |
| ADH020 | Healthy | skin folds |
| ADH023 | Healthy | skin folds |
| ADH024 | Healthy | skin folds |
| ADH025 | Healthy | skin folds |
| ADH026 | Healthy | skin folds |
| ADH027 | Healthy | skin folds |
| ADH028 | Healthy | skin folds |
| ADH030 | Healthy | skin folds |
| ADH031 | Healthy | skin folds |
| ADH032 | Healthy | skin folds |
| ADH033 | Healthy | skin folds |
| ADH034 | Healthy | skin folds |
| ADH035 | Healthy | skin folds |
| ADH036 | Healthy | skin folds |
| ADH037 | Healthy | skin folds |
| ADH038 | Healthy | skin folds |
| ADH039 | Healthy | skin folds |
| ADH040 | Healthy | skin folds |
| ADH041 | Healthy | skin folds |
| ADH042 | Healthy | skin folds |
| ADH044 | Healthy | skin folds |
| ADH045 | Healthy | skin folds |
| ADH046 | Healthy | skin folds |
| ADH047 | Healthy | skin folds |
| ADH048 | Healthy | skin folds |
| ADH049 | Healthy | skin folds |
| ADH050 | Healthy | skin folds |
| ADH051 | Healthy | skin folds |
| ADH052 | Healthy | skin folds |
| ADH053 | Healthy | skin folds |
| ADH054 | Healthy | skin folds |
| AD001 | mild AD | face |
| AD002 | mild AD | face |
| AD003 | mild AD | skin folds |
| AD004 | moderate-to-severe AD | skin folds |
| AD005 | mild AD | skin folds |
| AD006 | mild AD | skin folds |
| AD007 | mild AD | face |
| AD008 | moderate-to-severe AD | face |
| AD009 | mild AD | skin folds |
| AD010 | mild AD | foot |
| AD011 | mild AD | abdomen |
| AD012 | mild AD | skin folds |
| AD013 | mild AD | arms |
| AD014 | moderate-to-severe AD | abdomen |
| AD015 | mild AD | hands |
| AD018 | mild AD | back |
| AD019 | moderate-to-severe AD | arms |
| AD020 | mild AD | skin folds |
| AD021 | mild AD | legs |
| AD022 | mild AD | arms |
| AD023 | moderate-to-severe AD | skin folds |
| AD024 | mild AD | neck |
| AD034 | moderate-to-severe AD | skin folds |
| AD036 | mild AD | skin folds |
| AD047 | mild AD | skin folds |
| AD049 | mild AD | legs |
| AD052 | mild AD | skin folds |
| ADA001_A | mild AD | arms |
| ADA001_B | mild AD | arms |
| ADA002_A | mild AD | arms |
| ADA002_B | mild AD | arms |
| ADA004_A | mild AD | neck |
| ADA004_B | mild AD | neck |
| ADC002_A | mild AD | abdomen |
| ADC002_B | mild AD | abdomen |
| ADC003_A | mild AD | skin folds |
| ADC003_B | mild AD | skin folds |
| ADC004_A | mild AD | skin folds |
| ADC004_B | mild AD | skin folds |
| ADC005_A | mild AD | back |
| ADC005_B | mild AD | legs |

**Table S2:** participants’ demographics and information

| Total (n = 83) | | |
| --- | --- | --- |
|  | **[n]** | **[%]** |
| **Healthy participants** | **49** | **59.04** |
| - Gender female | 29 | 59.18 |
| - Antihistaminicum | 0 | 0 |
| - Topical corticosteroids | 0 | 0 |
| - Topical antibiotics | 0 | 0 |
| - Oral antibiotics | 3 | 6.12 |
| - Allergy | 3 | 6.12 |
| - Age range | 0 – 53 | / |
| - Mean age, stdv | 11.87 +/- 11.83 * | / |
| - 0-3 years | 11 | 22.45 |
| - 3-6 years | 10 | 20.41 |
| - 6-12 years | 9 | 18.37 |
| - 12-18 years | 10 | 20.41 |
| - >18 years | 9 | 18.37 |
| **AD patients** | **34** | **40.96** |
| - Gender female | 21 | 61.76 |
| - Antihistaminicum | 14 | 41.18 |
| - Topical corticosteroids | 14 | 41.18 |
| - Topical antibiotics | 3 | 8.82 |
| - Oral antibiotics | 3 | 8.82 |
| - Allergy | 8 | 23.53 |
| - Age range | 1-70 | / |
| - Mean age, stdv | 20.41 +/- 16.96 | / |
| **Mild AD patients** | **28** | **33.73** |
| - Gender female | 19 | 67.86 |
| - Antihistaminicum | 11 | 39.29 |
| - Topical corticosteroids | 10 | 35.71 |
| - Topical antibiotics | 2 | 7.14 |
| - Oral antibiotics | 2 | 7.14 |
| - Allergy | 6 | 21.43 |
| - Age range | 1-70 | / |
| - Mean age, stdv | 21.59 +/- 16.79 | / |
| - 0-3 years | 2 | 7.14 |
| - 3-6 years | 6 | 21.43 |
| - 6-12 years | 3 | 10.71 |
| - 12-18 years | 1 | 3.57 |
| - >18 years | 16 | 57.14 |
| **Moderate-to-severe AD patients** | **6** | **7.23** |
| - Gender female | 2 | 33.33 |
| - Antihistaminicum | 3 | 50.00 |
| - Topical corticosteroids | 4 | 66.67 |
| - Topical antibiotics | 1 | 16.67 |
| - Oral antibiotics | 1 | 16.67 |
| - Allergy | 2 | 33.33 |
| - Age range | 1-44 | / |
| - Mean age, stdv | 17.67 +/- 17.53 | / |
| - 0-3 years | 1 | 16.67 |
| - 3-6 years | 1 | 16.67 |
| - 6-12 years | 2 | 33.33 |
| - 12-18 years | 0 | 0 |
| - >18 years | 2 | 33.33 |

**Supplementary figures**

**
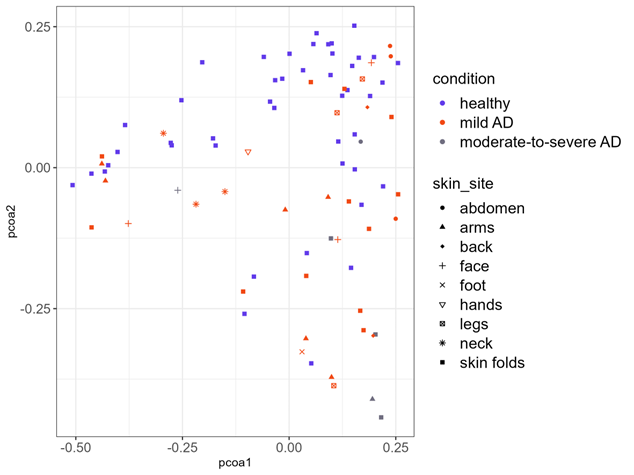
**

**Figure S1:** Principal Coordinates Analysis (PCoA) plot visualizing the beta diversity of AD skin samples, colored by skin condition and different shapes show the skin sites.


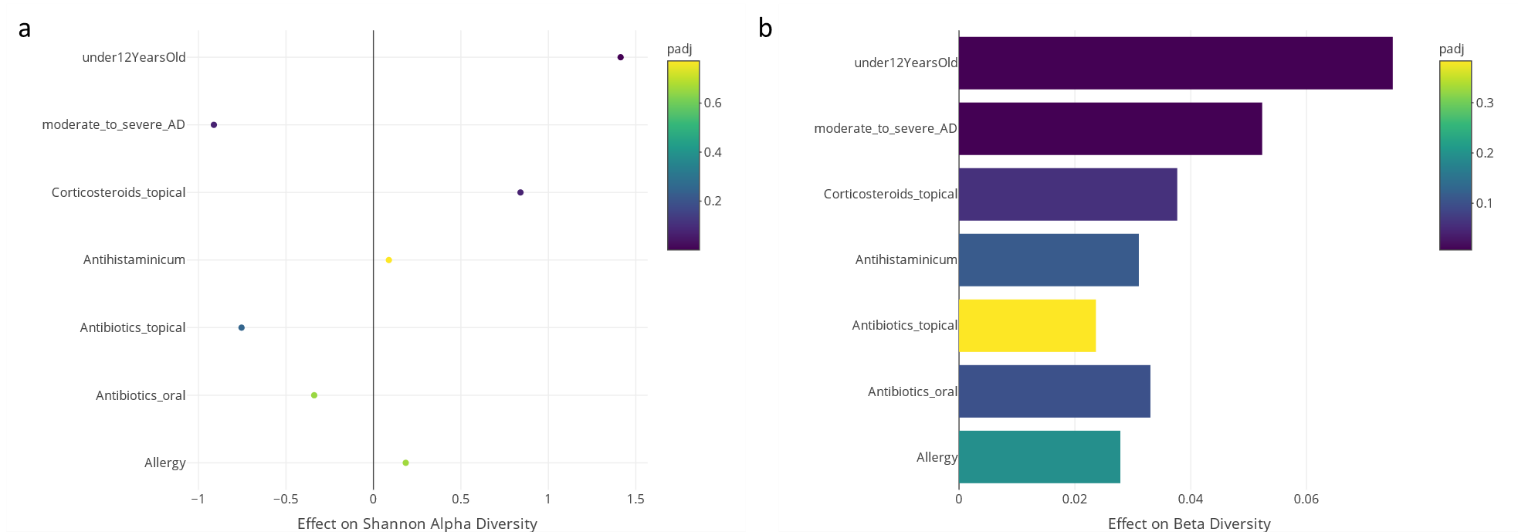


**Figure S2:** Effect of age and other host and environmental variables on the skin microbiome diversity in mild AD patients. (a) Alpha diversity calculated by Shannon index. (b) Beta diversity calculated by Bray-Curtis dissimilarity.


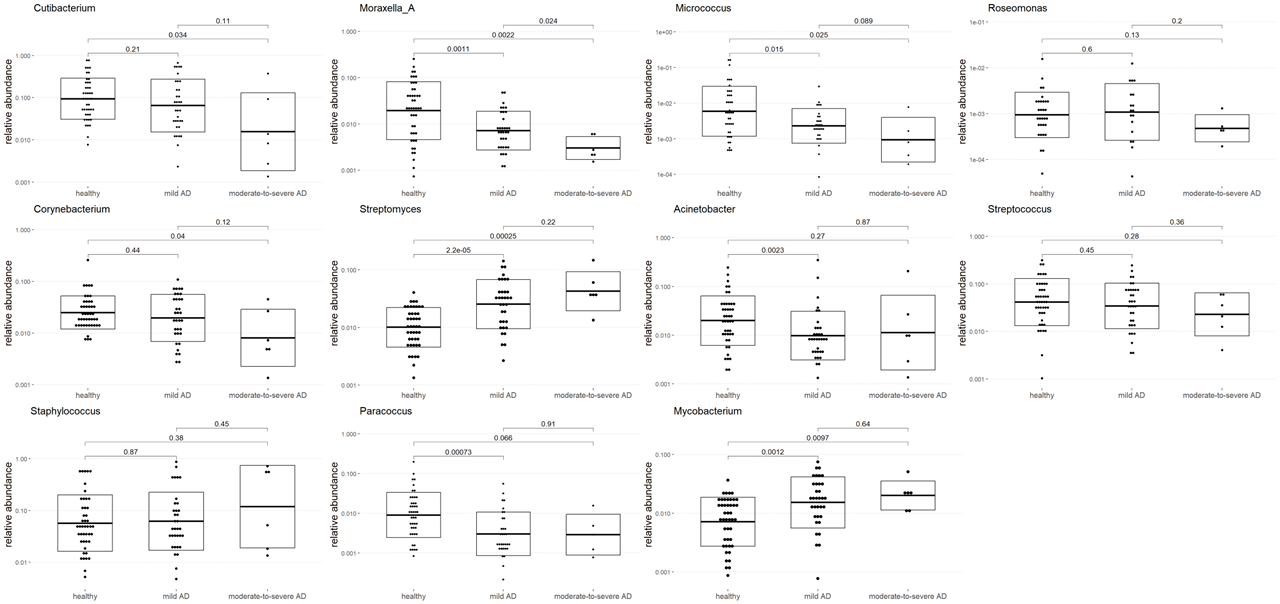


**Figure S3:** Boxplots showing the relative abundance of different skin genera in all three conditions, p-values are printed on the figures. Statistical differences between the groups were tested using Wilcoxon rank sum test.


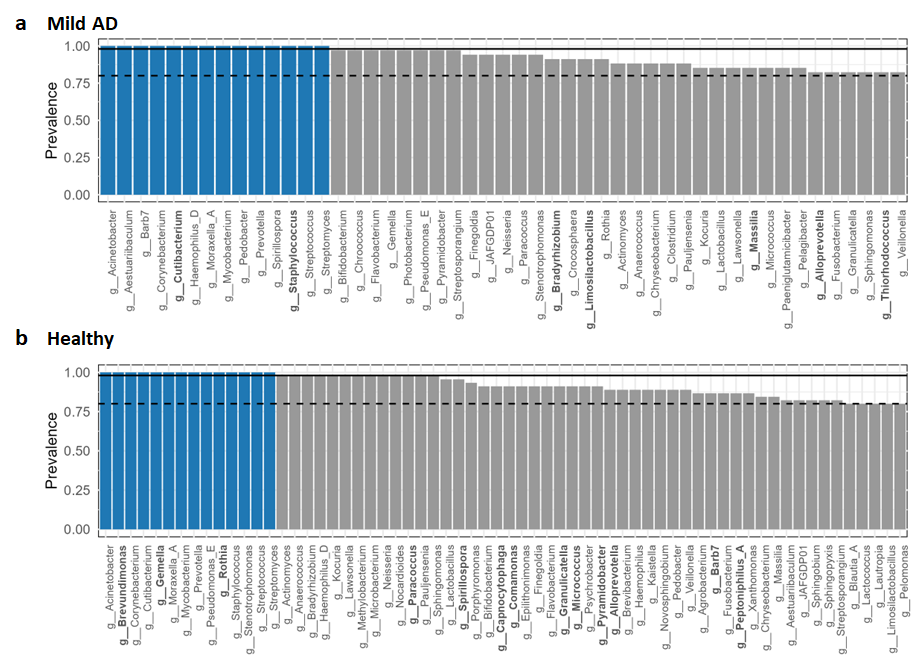


**Figure S4:** (a) Barplot showing the preservation of genera in the mild AD samples: all genera with a prevalence of at least 80% are shown as core prevalent genera. (b) Barplot showing the preservation of genera in the healthy samples: all genera with a prevalence of at least 80% are shown. The full and dotted line indicate a prevalence of 98% and 80% respectively. Blue represents omnipresent genera. Genera that were present for more than 98% in only one group, healthy or AD, are marked bold.


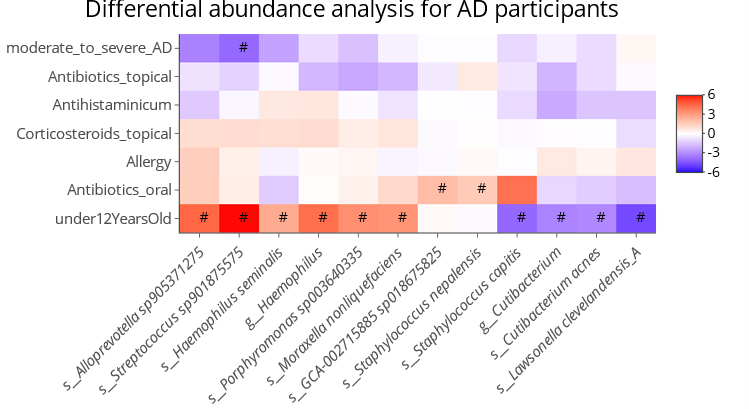


**Figure S5:** Differential abundance analysis of species for AD patients. A multiple linear regression with model rel_abund ~ AD severity + antibiotics + antihistaminicum + corticosteroids + allergies + age was tested using the default settings of the Maaslin2 package. The taxa displayed in the figure are those who have atleast one covariate with an adjusted p-value (BH) < 0.05.

**
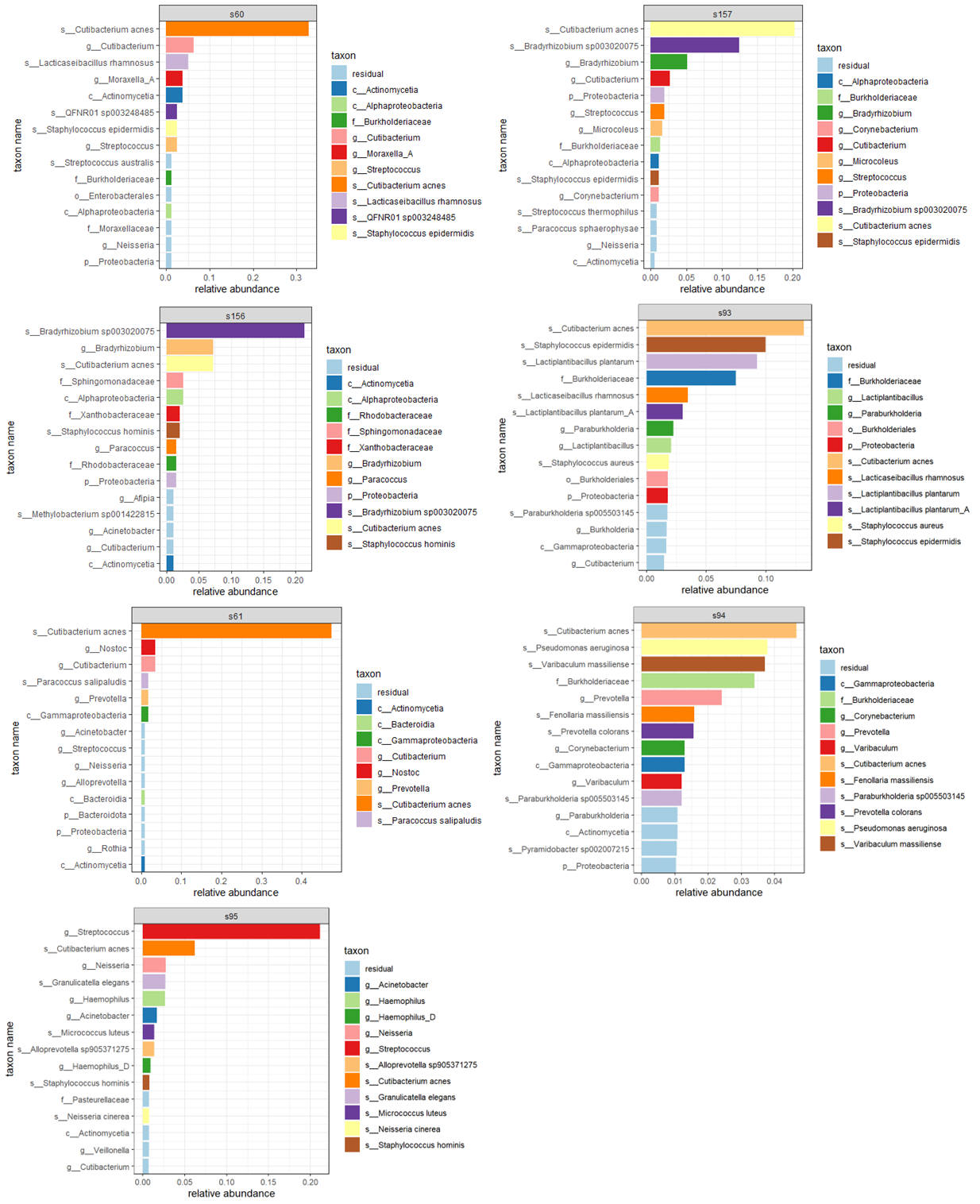
**

**Figure S6**: Relative taxonomic abundances of negative controls for metagenomic shotgun sequencing.

***
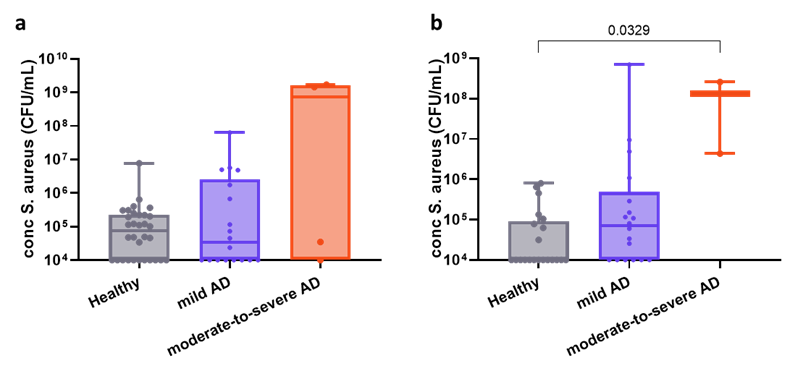
***

**Figure S7:** qPCR for absolute abundance of *S. aureus.* (a) All samples of participants below 12 years old. (b) All samples of participants of 12 years old and older. Statistics were performed with Kruskal-Wallis test with Dunn’s multiple comparisons test against the healthy samples. Significant p-values are indicated on the figure.
